# Climate change and adventure guiding: The role of nature connection in guide wellbeing

**DOI:** 10.3389/fpubh.2022.946093

**Published:** 2022-08-22

**Authors:** Elsa Valdivielso Martínez, Susan Houge Mackenzie

**Affiliations:** ^1^Department of Psychology, University of East London, London, United Kingdom; ^2^Department of Tourism, University of Otago, Dunedin, New Zealand

**Keywords:** adventure tourism, psychological wellbeing, sustainability, climate change, nature connexion

## Abstract

Ecological challenges are quickly shaping the future of the tourism industry with an increasing focus on how to develop more sustainable adventure tourism practises. Adventure guides play an important role in this transition and in shaping client experiences, however there is a need to better understand how climate change may have important impacts on guides' wellbeing. This study explored adventure guides' experiences of nature connectedness and potential links between climate change, nature connexion, and wellbeing for adventure guides. Semi-structured qualitative interviews (*x* = 11) with adventure guides were analysed using reflexive thematic analysis to explore these relationships. Adventure guides reported experiencing meaningful connexions and relationships with the natural environments in which they worked, while also highlighting why not all types of nature nor time spent outdoors facilitated this connexion. Guides that reported being more connected to nature also reported a higher sense of environmental responsibility, and guides described how this often created “ethical dilemmas” in seeking to resolve tensions between their deep connexion to nature and unsustainable practises that their guiding work often entailed. Analysis also highlighted the value and wellbeing guides derived from sharing their love of nature with clients. These findings expand emerging theoretical models of adventure guide wellbeing, and suggest a range of practises that can support a more ecologically sustainable adventure tourism industry.

## Introduction

Scholars have recognised the critical importance of developing a more environmentally responsible and sustainable adventure tourism industry in response to climate change^1^ [e.g., (1)]. Adventure guides' and their practises have been identified as playing a role in helping to mitigate the current ecological crisis *via* visitor education, interpretation, and sustainable travel practises [e.g., (3, 4)]. However, the primary focus of this literature has been on enhancing ecological sustainability practises of adventure tourists, operators, and guides, rather than understanding how climate change may impact guides' work experiences and wellbeing. Emerging literature has recently begun to consider guide wellbeing as a worthwhile research focus in and of itself, rather than simply a means to enhance visitor satisfaction [e.g., (5)]. These studies have highlighted the central importance of connexion with nature in guide wellbeing, alongside other intra and interpersonal factors [e.g., (5, 6)]. Nevertheless, there is a need to expand this literature to better understand various dimensions of adventure guides' relationships with nature and how this relationship relates to their wellbeing and work experiences. Specifically, studies have yet to directly explore relationships between climate change and adventure guides' experiences of nature connexion and wellbeing. Research on these relationships is timely and increasingly important given that (1) climate change is fundamentally affecting natural landscapes globally at an accelerating pace, and (3) the global tourism is changing dramatically as a response to climate change. The current study sought to address these knowledge gaps by exploring adventure guides' experiences of nature connexion and wellbeing in relation to climate change.

## Literature review

This literature review begins by defining the concept of adventure tourism and contextualising adventure activities in relation to participation trends and associated wellbeing benefits. Research on adventure guide experiences is then discussed alongside literature highlighting the significant role that the natural environment plays in this profession. This section concludes with a presentation of findings related to adventure guide wellbeing and nature connectedness, and the associated rationale for applying an eco-psychology lens to understand relationships between climate change, nature connectedness and adventure guide wellbeing. It is important to note that, while the primary focus of the current paper and literature review is on guide wellbeing (i.e., human wellbeing), ecopsychology perspectives recognise that the various wellbeing concepts discussed below can be more broadly examined in terms of non-human wellbeing, and that guide wellbeing also has implications for the wellbeing of natural environments.

### The adventure tourism context

Defining “adventure tourism” has been a matter of much academic discussion. Not only has this sector changed enormously in the last few decades, transforming from a niche tourism offering to a wide range of accessible, commodified forms of tourism (7, 8), but adventure tourism conceptualisations have also evolved. Earlier conceptualisations of adventure tourism mainly focused on physical characteristics, whereas more recent approaches have had a greater focus on psychological characteristics and experiences. In addition, while adventure tourism shares some characteristics with “ecotourism,” it is considered a distinct sector as ecotourism has been more clearly defined in terms of the following key characteristics: nature-based products, minimal impact management, environmental education, contribution to conservation and communities, ethics/responsibility, and sustainability [e.g., (9, 10)].

In an effort to better differentiate adventure tourism, Rantala et al. (11) conducted a systematic literature review and concluded that adventure tourism has different meanings depending on the context and is often used as an umbrella concept rather than as an analytical one. Although various scholars have highlighted physical activity, the natural environment and risk and danger as core characteristics of adventure tourism [e.g., (12)], one of the largest global adventure tourism industry associations, the Adventure Travel Trade Association (ATTA) uses a broader definition. Adventure travel trade association (13) proposes that adventure tourism, “contains three main components for the traveller: (1) physical activity; (3) a connexion to nature and the environment; and (4) an immersive cultural experience” (p. 2). As this industry-based definition can be critiqued as being overly broad and simplistic, or omitting the core distinguishing factors of adventure tourism, Janowski et al. (12) offered a more comprehensive understanding of adventure tourism based on a recent systematic review of the adventure tourism literature. Their conceptualisation proposed the following key components that differentiate this form of tourism from other types: *risk and danger, the natural environment, thrill and excitement, challenge, and physical activity* (12). Although this conceptualisation encapsulates many of the commonly cited dimensions of adventure tourism, academic consensus on this term remains elusive (12).

In economic terms, adventure tourism has been identified as a growing industry, and its global market value has been projected to increase from $852.4 billion in 2021 to 2,548.2 billion in 2027 (14). To better comprehend this rise, scholars have sought to understand why people are increasingly participating in adventure travel. It has been suggested that Western lifestyles, characterised by safe and sanitised ways of living disconnected from nature, or what has also been called “nature-deficit disorder” (15), have motivated people to seek new ways to define their lives and identities [e.g., (16, 17)]. For example, Lee and Ewert (18) proposed that adventure activities have the potential to help people develop personal and social identities that are more attractive than those resulting from conventional careers and lifestyles. In addition, this form of tourism makes tourists feel unique and distinct from tourists who participate in ‘mass tourism' activities (19).

Traditionally, adventure tourism scholars have also highlighted thrill-seeking as a key motivation underpinning tourists' desire to participate in guided adventure activities [e.g., (20)]. This perspective aligns with traditional portrayals of adventure participants in the literature as self-focused, adrenaline-seeking hedonists [e.g., (21)]. However, recent adventure literature has proposed alternative understandings of adventure motivations, which focus on the broader wellbeing benefits for participants –[e.g., (16, 22, 23)]. These wellbeing benefits have been associated with opportunities to have unique experiences, build competence, and connect with others, as well as opportunities to connect with unique natural environments [e.g., (3, 6, 24)].

### Adventure tourism and the natural environment

Despite the range of potential benefits that adventure appears to offer participants, there is an unresolved tension in the literature regarding the environmental costs and benefits of adventure tourism. Some authors argue that, regardless of the wellbeing benefits, adventure tourism is unsustainable due to the impact it has on fragile environments [e.g., (25, 26)]. For example, the travel required to access some of these environments creates high levels of carbon emissions, which exacerbates the impacts of climate change on the environments tourists seek to experience [e.g., (27)]. In contrast, other scholars suggest that the adventure tourism industry can be environmentally sustainable by fostering emotional attachments to nature and spiritual experiences of awe and transformation [e.g., (28)] *via* direct involvement with natural environments (3, 29), which is critical to promoting sustainable, pro-environmental behaviours [e.g., (30)]. Knowles (28, p. 102809) offered a nuanced perspective on this tension by arguing that,

Adventure tourism is not in itself sustainable but by targeting adventure travel that attracts passionate, high-paying tourists to participate in activities specifically suited to local landscapes, communities have the potential to create bespoke tourism products that fit the socio-ecological system and produce clear conservation and sustainable development outcomes.

Hanna et al. (3) concur that adventure has the potential to be a sustainable form of tourism by facilitating pro-environmental attitudes and behaviours that benefit non-human nature, while also supporting human health and wellbeing.

This literature highlights a range of potential benefits for people and places associated with adventure travel. However, the adventure tourism literature has been largely tourist-centric in that it has primarily focused on tourists' experiences and behaviours, or the role of guides or products in tourist satisfaction outcomes. This focus has left the experiences of adventure guides relatively underexplored, particularly in relation to wellbeing and sustainability issues. Thus, the following section will explore the emerging literature on adventure guide experiences.

### Adventure guiding and wellbeing

Before exploring the literature on adventure guide experiences, this section will contextualise adventure guiding by briefly outlining guide roles and responsibilities. Guiding is complex as guides not only need to possess excellent technical skills to manage the risk involved in these activities and guarantee their clients' safety (31), but also need to become social facilitators, environmental interpreters, inclusive leaders, pathfinders and mentors [e.g., (4, 32–34)]. Moreover, tourists, especially those with access to communication technologies (e.g., Facebook, YouTube), now increasingly seek purposeful and meaningful tourist experiences, rather than experiences that are simply “fun.” Thus, some scholars have suggested how guides can adapt to these new trends and move away from being a “one-way presenter” towards “co-created” approaches that adapt to clients' emerging demands and seeks to co-create unique guided experiences (4). This approach entails a broader range of guiding roles and skills, such as brokering empathy and cultural understanding for people and places, facilitating tourists' self-development [e.g., (35)], and developing sustainable tourism practises in adventure contexts [e.g., (29)].

A growing body of literature suggests that the occupational demands associated with adventure guiding are distinct from those expected of their counterparts in other tourism sectors. Adventure guides are required to dynamically manage highly challenging physical and cognitive tasks, as well as the emotions and safety of their clients and co-guides in changeable natural environments (36). There are several features that distinguish adventure guiding from other forms of tourism work. For instance, adventure guides are required to embody the excitement they are selling in order to fulfil their clients' expectations (37). However, showing negative emotions, such as anxiety or fear, is often considered unacceptable to clients unless they are in imminent danger (32, 38, 39). Guides not only need to manage their own emotions, but they also have to manage their clients' emotional experiences [e.g., (36, 40)]. As a result, some adventure literature has begun to explore guides' emotions and intrapersonal experiences, not simply the tourist impacts of guide experiences [e.g., (32, 40, 41)]. Similarly, Carnicelli-Filho (32) identified that, in adventure guiding roles, “work” and “non-work” emotions merged. In other words, guides often consider their work to be not simply a profession, but rather a way of living. In addition to literature highlighting the unique emotional nature of adventure guiding, research has also begun to holistically explore psychological approaches to understanding adventure guide wellbeing and experiences [e.g., (23)]. For example, it has been suggested that adventure guiding can be characterised by high levels of job satisfaction (42) through experiences of authenticity (40) and deeper meaning (43).

Notwithstanding this new turn in tour guiding research, the findings are mixed in terms of how adventure guiding impacts wellbeing. In addition to documenting wellbeing impacts, the literature has also documented the emotional labour and ill-being effects of these challenging roles [e.g., (36, 40, 44, 45)]. As a result, studies have reported how adventure guides often use mental practises such as “deep acting” and storytelling to decrease emotional labour and enhance the perceived meaningfulness of their roles (43, 46). Houge Mackenzie and Raymond (5) attempted to account for these mixed findings by proposing a conceptual model of the key psychological wellbeing determinants for adventure guides by drawing on basic psychological needs theory [e.g., (47)]. These authors found that the degree to which guides experience a sense of competence, autonomy, relatedness, nature connexion, and beneficence (positively impacting others) largely determined whether or not guides derived wellbeing from their guiding roles. For instance, women adventure guides reported frequently receiving feedback from clients, co-guides, or managers that suggested they were less competent than male guides, which thereby decreased their wellbeing in this context (6). Collectively, this literature suggests that adventure guiding has the potential to support guide wellbeing *if* these roles support key psychological needs, such as feelings of competence, creating a positive impact, and a sense of connexion to the natural environment.

The following two sections provide a deeper theoretical perspective on these findings related to adventure guide wellbeing and discuss how these findings relate to the current study. First, key concepts in the emerging literature on adventure guide wellbeing are discussed, followed by a section outlining links between adventure guide experiences, nature connectedness and ecopsychology. Subsequently, the current study aims and research questions are presented.

### Adventure guide wellbeing

A number of psychological constructs have been proposed to determine adventure guide wellbeing, such as the basic psychological needs theory constructs of autonomy, competence and relatedness [e.g., (47)]. Houge Mackenzie and Raymond (5) suggested that, beyond guide needs for autonomy, competence and relatedness, the ability to have a positive impact and connect with nature may also be key wellbeing determinants. However, this framework has yet to be empirically tested and a more comprehensive conceptualisation could be considered. For instance, few psychological models consider nature connexion, as defined in the following section, is a fundamental part of wellbeing, despite robust findings regarding the benefits of various forms of nature contact and connexion (e.g., urban vs. wild nature) (48–52). Studies have shown, for example, the positive impact of encounters with urban nature on people's wellbeing regardless of age (53, 54). However, recent research has also suggested that wild nature and biodiversity have stronger correlations with human wellbeing (55, 56). These findings suggest that adventure guiding has the potential to increase a sense of connexion to nature, and thereby to enhance wellbeing as adventure activities generally unfold in more natural areas.

For adventure guides, who are extensively exposed to nature as part of their everyday work, understanding how the natural environment influences their wellbeing is critical. This issue is particularly relevant given the current environmental crisis (57). While nature contact has been identified as a key determinant of wellbeing in the adventure context, no studies have yet considered the flip side of this proposal: how the degradation of the natural world may potentially detract from the wellbeing of adventure guides.

### Eco-psychology, nature-connectedness and adventure guiding

The field of eco-psychology explores human-nature relationships and offers a perspective on wellbeing that is different from traditional psychological approaches (58). Eco-psychology seeks to understand human-nature relationships beyond traditional dualistic stances that view nature as separate from human beings (59). This perspective embraces a shift away from more “anthropocentric” (i.e., human-focused) approaches, towards more “eco-centric” perspectives [nature-focused, viewing humans as attached to, not separate from, nature; (60, 61)]. Eco-psychology focuses on how human and planetary health influence one another and posits that environmental challenges result from broken relationships between humans and nature (58, 62). In recent decades, there have been an increasing number of eco-psychology studies that have aimed to better understand the complexities of the relationships between human wellbeing and nature. For example, some research has outlined the benefits of exercising in nature (e.g., “green exercise”) in relation to both human wellbeing as well as a means of protecting natural environments and biodiversity *via* developing connexions with nature [e.g., (63)]. In line with these findings, nature-based interventions, such as gardening, have been found to reduce stress, foster meaningful social relationships, and enhance relationships with all beings, not just amongst humans [e.g., (64–66)].

Despite the documented benefits of nature for wellbeing, industrialised societies have become increasingly disconnected from it [e.g., (67)]. For instance, Louv (15) coined the term “vitamin N” to express the deficits associated with the lack of exposure to nature in modern societies. In recognition of this issue, doctors in some countries (e.g., UK, New Zealand) have started prescribing nature (e.g., “green prescriptions”) to improve a range of pathological and non-pathological conditions (55). However, Richardson et al. (51) highlight that it is not the time spent in nature, but the *quality* of this time that benefits individuals. For instance, actively attending to short tasks, such as smelling flowers, may be more beneficial than a long walk in the woods if an individual is engaged with their environment during such activities.

The quality of nature experience appears particularly relevant in relation to understanding wellbeing impacts of nature for people and environments. Simply being in nature is different than feeling connected with nature. This recognition has given rise to the analogous constructs of nature relatedness and nature connectedness [e.g., (68, 69)]. Nature connectedness is an individual's subjective sense of their relationship with the natural world (70), and has been defined as “the extent to which an individual includes nature within his/her cognitive representation of self” (69, p. 67), or, in other words, the extent that individuals feel one with the natural world (71). This conceptualisation fits within the eco-psychology approach as it extends beyond mere exposure to nature by highlighting how nature connexion relies upon incorporating nature within the self-concept (72, 73). For instance, Pritchard et al. (52) found that individuals who felt connected to nature in this way had a higher sense of psychological wellbeing and lower levels of ill-being (i.e., depression and anxiety). These findings lend support to scholars who have argued that the current conceptualisation of ‘relatedness' should be expanded beyond human relationships to include human-nature relationships, by adding ‘nature-relatedness' (74) as a basic psychological need. In addition to being associated with human health and wellbeing, nature connectedness has associations with pro-environmental behaviours, which benefit non-human nature also (75).

If nature relatedness has the potential to be a basic psychological need, then understanding the type of nature exposure that enables connexion to nature is critically important. By extension, the crises facing the natural world (e.g., loss of biodiversity and wild places), may also pose psychological risks to individuals if they lose the ability to feel connected to nature. For instance, individuals who endorse high levels of nature relatedness may be at high risk of experiencing psychological ill-being due to climate change (76). These experiences are captured by a range of related constructs such as *solastalgia* [i.e., distress produced by environmental changes impacting people's connexion to their home environments; (77)]; *eco-anxiety* [i.e., anxiety that occurs as a result of the ecological crisis; (78)]; and similar terms such as *eco-guilt, eco-depression*, and *eco-grief* [i.e., a range of negative emotions derived from the consequences of climate change, (79)].

Adventure guides' wellbeing has been shown to be linked to nature connexion [e.g., (5)] and many ski, snowboard or glacier guides, witness the impacts of climate change on a daily basis (57). For example, Salim et al. (80) documented how mountain guides are adapting their daily practises based on climate change. Notwithstanding, little attention has been paid to understanding the psychological impacts of climate change on adventure guides. While previous studies have explored the important role of contact with nature in guiding (5), no research has explored how guides' wellbeing is influenced by environmental changes due to climate change (81), environmental degradation (82), and/or the commodification of wild places (83). Therefore, investigations of guide wellbeing that directly consider their connexions to nature in the context of climate change are needed.

### Study aims and research questions

The current study sought to better understand adventure guides' relationships with nature and how this relationship relates to their wellbeing and work experiences in the context of climate change. This investigation was guided by the following research questions:

1. How do adventure guides experience nature connexion, and how is this relationship linked to their wellbeing?
2. How is climate change impacting adventure guides' experiences of nature connexion and wellbeing?

## Methodology

### Research design

The current study was guided by critical realism, which embraces ontological realism, but posits that scientific enquiries are interpretive in nature [i.e., epistemological constructivism; (84)]. Thus, researchers can only generate incomplete understandings of the world (85). Critical realist research is compatible with theoretical frameworks, however critical realist researchers acknowledge that theoretical accounts of the world are necessarily incomplete and fallible (86). As the current study was seeking to better understand complex subjective experiences, an exploratory qualitative research design was employed to develop a better framework for understanding guides' experiences and wellbeing in relation to climate change [e.g., (84, 87)].

### Participants and sampling

The lead researcher recruited participants *via* social media channels using purposeful and snowballing sampling (88, 89). Guides were purposefully sampled on the basis of having worked in the adventure guiding profession for at least 1 year. The lead researcher posted a notice about the research and participants were sought on adventure-related social media sites (e.g., Facebook, Twitter). Guides who expressed an interest in participating in the study were then sent a formal invitation email with full study and consent information.

The final sample consisted of 11 adventure guides (7 men, 3 women, 1 gender undisclosed; mean age = 43 years, *SD* = 10 years), who worked across a range of land and water-based adventure activities (e.g., mountain leading, kayaking, canyoning, rock climbing; see Table 1 for complete list of activities). Participants were from Britain (*n* = 7), Greece (*n* = 1), Portugal (*n* = 1), Ireland (*n* = 1), and France (*n* = 1). Although researchers sought to capture a range of different adventure guiding perspectives, the sample was male-dominated, which reflected the demographics of adventure tourism guiding industry [e.g., (6)].

**Table 1:** Participants demographics.

**Name**	**Age**	**Gender**	**Nationality**	**Main guiding activities**
Susan	34	F	British	Mountain leader
Isa	37	U^*^	Greek	Mountain leader, kayaking guide
Izabella	45	F	British	Mountain leader
Evan	62	M	British	International mountain leader
Emma	48	F	British	Sea kayaking guide
Leo	40	M	Portuguese	Canyoning guide
Callum	33	M	Irish	International mountain leader
Liam	54	M	British	International mountain leader
Daniel	58	M	British	Sailing guide
Kevin	34	M	British	Canoeing, mountain leader
Elliot	32	M	French	Canyoning, rock climbing guide

* Undisclosed gender.

All names are pseudonyms.

Ethical approval for this study was granted by the [redacted for blind review] Ethics Committee. Before each interview, the lead researcher provided participants the study information and consent forms, explained to the participants the confidential nature of the study and their right to withdraw from the study at any time, and obtained consent prior to the start of each interview. After each interview, a debriefing letter was also sent to participants.

### Data collection

Data were collected through online semi-structured interviews to allow guides to participate at their convenience (90). Data were collected over a 2-month period with interviews lasting from 33 to 65 min (mean length = 49 min). The interviews were held virtually through Microsoft Teams software and were recorded using the built-in screen recorder option. Microsoft Teams provided a basic transcription of the interviews, which was used as an initial base for manual transcription. All interviews were transcribed verbatim. To ensure participant anonymity, all guides were given pseudonyms.

#### Reflexivity

To ensure the interview guide was appropriate to address the study aims, a pilot interview was conducted [e.g., (91)] with an adventure guide who was external to the sample. As a result, the interview was refined based on their feedback (e.g., some questions were rephrased or removed from the interview guide). Despite these revisions based on the pilot interview, the lead researcher identified an additional question which had the potential to elicit leading responses in her initial interviews. After discussing this concern with an experienced researcher external to the study, this interview question was rephrased to eliminate this issue.

The revision process described above reflected one example of how researchers engaged in reflexivity at each research stage. The lead researcher reflected throughout the research process on how her insider knowledge of adventure guiding and background could potentially influence the research process and findings (92). Although the lead researcher did not have a professional guiding background, she participated in adventure sports and nature-based recreation on a regular basis and had a strong affinity for nature. The supporting researcher had a professional guiding background, as well as an academic background in psychological wellbeing and qualitative research and reflexive processes. The researchers' backgrounds benefitted in the study in relation to their awareness of reflexive processes and how values and perspectives influence research processes, as well as enabling the lead researcher to establish rapport quickly and easily with participants. However, throughout the research process the lead researcher was sensitised to how her values and perspectives on nature, climate change, and sustainability could influence the research process. To ensure she remained aware of, and reflexive to, these influences, she closely monitored and reflected on her values and perspectives throughout the research process by engaging in weekly journaling and research discussions with peers and advisors (91).

### Data analysis

A reflexive thematic analysis process (93) was used to analyse the data. As reflexive thematic analysis does not narrowly stipulate data collection processes, nor does it necessarily reflect a specific epistemological or ontological framework: it is a method that is compatible with various epistemological approaches (93). In the current study, the researchers adopted a critical realist approach, which views knowledge as socially influenced (94, 95). The researchers recognised that the world exists independently of our knowledge of the world, therefore this research considered adventure guides' experiences and perspectives in relation to both existing knowledge models of adventure guide wellbeing (5) as well as in relation to guides' idiosyncratic perspectives reported in the data (85).

Braun and Clarke (91) recommended six key stages of data analysis when using thematic analysis: familiarisation with the data, coding, generating initial themes, reviewing themes, defining and naming themes, and writing up the findings. In the current study, these phases were followed in an iterative, rather than linear manner, to allow for reflexivity, flexibility and continual revisiting of themes and concepts within the data (93). To immerse herself in the data, the lead researcher first re-read each transcript multiple times and then conducted an initial manual analysis of the data. After this process, each interview transcript was imported into the qualitative software management tool Nvivo to help the researcher manage codes and generate themes, and to revise codes and themes through iterative stages of analysis. The codes were assigned at both semantic and latent levels. At a semantic level, some codes were more descriptive and concrete as they described guides' experiences of nature connectedness. At a latent level, the researcher assigned different codes that reflected higher-level conceptual interpretations of the data. The codes were then clustered together, and the lead researcher generated initial candidate themes.

In the next phase of analysis, the lead researcher returned to a manual analysis approach to facilitate a different way of looking at the data and candidate themes, and to identify any themes that could be collapsed (96). In this stage, manual visual-mapping was used, which allowed the lead researcher to identify overarching patterns in the data (91). The visual-mapping process allowed for more refined themes to be generated. These refined themes were again revised by rereading the verbatim transcripts, and referring back to the literature, to ensure congruency between the themes and participants' raw quotes (91). In the last stage of analysis, Nvivo was again used to organise the final themes and subthemes alongside representative quotes.

## Results and discussion

The aims of this study were to (1) understand adventure guides' experiences of nature connexion; and (3) explore potential links between nature connexion, climate change and wellbeing for adventure guides. The following section integrates the results and discussion in relation to each of the three primary themes identified in the data: *nature (dis)connexion, dare to care*, and *sharing nature connectedness with others*. Each theme is first defined and explained with reference to representative quotes, and then discussed in relation to the literature.

### Nature (dis)connexion

Participants in this study reported relating to nature in two opposing ways. Paradoxically, all guides reported viewing themselves and their guiding activities as distinct and separate from nature (i.e., an anthropocentric perspective characterised by a human-nature dichotomy), while all guides, with one exception, also reported an opposing perspective in which they felt they were a part of, and belonged to, nature (i.e., an eco-centric perspective) (61).

From an anthropocentric perspective, participants often referred to nature as something to either fear, use, or admire for its beauty. For some guides, being in nature evoked feelings of fear and intimidation*: “[My work environment is] a riverbed, so pretty wild and could be a little bit scary sometimes … it's noisy … it's intimidating … [especially when the] weather is not very good. [The canyon] could be hostile”* (Elliot). Similarly, another guide reported being overwhelmed and scared, especially when guiding in harsh environments: “*[There are times when you are] in the middle of the desert, you see a bird circling around above your head and you're like ‘is he waiting for me to [fall] over and die?' … 'cause it's a brutal environment the desert, it's harsh”* (Kevin).

Nature was also characterised by some participants as something to use for their own pleasure: “*Before I went kayaking, I didn't realise that these [wild and remote] places existed, that you can actually use them. For a night or two they're your home, it's amazing”* (Emma). In addition, one participant reported using nature as a playground for her own entertainment and referred to the importance of guide's using nature to match their clients' physical capabilities, and to make the guiding experience more enjoyable: “*In the outdoors I basically have a huge playground to go and explore … [and with clients] it's [about] using the environment in the right level of physical challenge”* (Susan).

The natural environments guides worked in were also described as something beautiful to be admired and contemplated. For example, Daniel reported this in relation to wildlife encounters: “*When the dolphin comes up and pops his head out of water and you can see its eyes looking at you … that's about as close as you can get I think to nature, other than sitting on top of a big mountain admiring this huge great big [landscape], thinking you're the only person there.”* Another guide also reported being astonished by the beauty of natural places: “*One of the phases of this expedition was trekking in this national park (…) and I found myself on top of this mountain and … I was absolutely blown away [by the beauty of the place]”* (Liam).

The quotes above highlighted how guides' experiences of nature were multifaceted and varied, ranging from fear to admiration, as well as more utilitarian connexions. Despite these descriptions that reflected a sense of separation between guides and nature, all participants except one reported a strong sense of connexion, or ‘oneness' with nature, which reflected a more eco-centric point of view. For instance, one guide expressed a profound sense of connexion with nature facilitated by his deep understanding of the natural environment*: “When you [work in the desert] and to be able to read [and understand] the sand like that … that's amazing … you feel really connected with nature. At the time … it was like a Eureka moment in my life”* (Kevin). Another guide experienced this as a sense of belonging to nature: “*I've probably worked outdoors for so long now that it just feels like my natural habitat, it's where I belong”* (Izabella). Callum also reported experiencing this feeling of ‘oneness' when immersed in nature while guiding:

*[When you are in nature] you can't hide and there isn't concrete covering the natural world around us … it feels very connecting because there's less barriers between ourselves and each other and ourselves and the world … if it's cold, you feel the cold. You can't just flick a switch and turn on the heating … it represents a much higher version of connexion*.

However, not all types of nature facilitated this sense of connexion. This finding aligned with previous research suggesting that the level of wilderness in a place influenced the meaningfulness of the interaction with nature (56). For instance, some guides reported varying degrees of nature connexion depending on its degree of wilderness and biodiversity. Isa explained how a particular type of wild nature heightened the feeling of belonging to it: “*I love wild nature … I can … just feel it … It's not something superficial … my senses are more open and I can feel … it's wild, it's not just a forest in the suburbs of a city … [When I'm in wild nature] I'm more myself.”* Elliot also reported how a lack of wilderness and biodiversity not only inhibited these feelings of connexion with the environment, but was currently decreasing his desire to continue guiding: “*nature for me means wilderness and biodiversity … when you see many people at the same place it destroys everything [you] got less animals you got less … calm and less wild [so if it keeps this way] I won't be as motivated [to guide].”*

Expedition length, as well as the physical effort required and activity type, were also reported to influence guides' feelings of connexion with nature. Longer expeditions, for example, were reported to contribute to a deeper nature connexion. Below, Callum described how these powerful experiences facilitated nature connexion:

*I feel more connected [to nature]…[when] we've had a couple of pretty difficult travel days … and then we get a day where we get to where we aim too early and … there's just this moment [of connexion]. I think it's partially because you know you're exhausted … but there's just this moment of: we're sort of very free and we're very here… you know if you're lucky enough where it's sunny and things are comfortable, you just sort of feel like the world is very balanced and it wouldn't feel the same had we not done the work, but it also wouldn't feel the same if we weren't in this outdoor setting. If we just walked into a comfy house, there would be a feeling of relief, but it wouldn't be as powerful…I guess immersion in some form…[and] the fact that it's all around you, there's something about it*.

Notwithstanding, not all time spent in nature was reported to facilitate feelings of nature connexion. For instance, several guides reported finding it easier to enjoy nature when they did not have the responsibility of guiding clients. For instance, some guides explained how being in nature with clients could create a more superficial connexion at times, as her attention was diverted towards clients' safety, as exemplified below:

*Often with clients I'm waiting … for them all the time which is … normal but I don't often get a chance [to focus] on me and what I'm doing … when I'm working I'm thinking about how are [clients] feeling [and] I'm focusing on them rather than the sea* (Alice).

Isa reported similar experiences; they found it easier to connect with nature when exposed to it by themselves or with friends, rather than while guiding:“*[I worked] mostly in the weekends, during the week [I was] going into the forest [to] hike by myself or just with friends … so I had the time to spend real time with nature, not just … as a guide.”*

Although there is spare literature on guides' experiences of nature connexion in both guided and non-guided contexts, these findings extend previous research suggesting that it is not the amount of time spent in nature, but rather the *quality* of this time that enhances wellbeing (51).

Given guides' deep connexions to nature, it was unsurprising that many study participants reported being closer to nature was a primary motivation to begin guiding: “*I think that I chose the mountain guiding … just to be closer to nature as much as possible*” (Isa). In addition, working as a guide was also considered to be a way of spending more time outdoors: “*Being out in nature … is my place to be really so that then got me thinking … how can I get out into the outdoors and every day? so doing it as a [guiding] job [was the answer]”* (Susan). Nevertheless, the enjoyment of guiding was also influenced by the type of environment where the guiding experience occurred. For example, what one of the participants appreciated the most about guiding was the possibility to be in isolated areas: “*[As a guide] I love being somewhere you know where there's peace and quiet and maybe just you and your little group and get into really remote places”* (Liam).

### Dare to care

The deep, emotive relationships that guides reported having with nature was also reported to facilitate a sense of care for nature, as Callum explained: “*The world is all so connected. I think it's hard not to have sort of a global idea of things and … [a] global idea of … ‘well it's all mine'... [but] how much is mine is related to how much I care about it”* (Callum). Similarly, Izabella reported caring for nature as a result of the immense value nature had for her, not just at a professional level, but also on a personal level. “*Aside from my work I have a strong personal connexion to being out in nature … so I'm really passionate about it and I really care about it … it's a place that's really precious to me*.”

Although participants expressed a profound connexion to nature and, as outlined above, they reported a sense of care for it, concern for the fate of nature in the face of ecological disasters and climate change was also reported. Liam, for example, described how he witnessed the deterioration of natural places: *Local people … were saying that … their crops are failing … and, although they didn't say, you know, it's to do with climate change you could, you can tell … it is making a difference and it's real, you know, for anyone who says it's not, it's real for sure. I've seen it*.

This quote illustrated the deep level of climate change awareness expressed by some guides. However, interestingly, the way guides related to climate change and the emotions experienced as a result were quite varied. For example, some guides experienced guilt: “*I obviously value the environment, but I'm part of the problem if I take these long-haul flights all the time and I visit these wonderful places only to add to the problems*” (Evan). Another guide expressed being upset: “*It's not a case of I'm annoyed at others or whatever like you'll hear the news or you'll see something [about climate change] and it will upset you and generally, unfortunately, it is upsetting*” (Callum). Conversely, other participants had not deeply considered the effects of climate change in relation to their guiding or wellbeing: “*does [climate change] affect my mood? I don't know. I've never really thought about it like that … Yeah, I don't know*” (Kevin). There were also guides who reported being cognitively aware of climate change, but who remained emotionally unaffected by it: “*I'm not a hundred per cent sure that [climate change] affects my mood. I think I might be aware of it. I don't think [it] actually affects [my mood in] what I do*” (Daniel).

While not all guides felt emotionally affected by climate change, those who did reported feeling an ethical dilemma in relation to their love for nature and the desire to transmit this love, and the environmental impact of their guiding practises. As Callum explained,

*I was having some ethical dilemmas because part of what I do is based on the fact that I love the outdoors and would like other people to love it and enjoy it. However, I could take five, six, seven … intercontinental flights a year, which is possibly one of the most damaging things I could do*.

Some other guides experienced this tension between their work and climate impacts, as a dissonance between their love for nature and their environmental consciousness. For instance, Susan reported her love of the natural world and how this supported wellbeing: “*I love … nature for me and my own mental health and wellbeing, and love sharing it with other people*.” However, she also reported an awareness of hypocrisy in relation to her passion for the environment and her contributions to environmental change: “*I would like to say that Covid has sparked off some big environmental consciousness [and that] I won't fly anymore, but it's not true. I will as soon as I'm able. I will definitely start getting on foreign expeditions again*.” This finding is consistent with previous research that explored the attitudes of air travellers in relation to climate change. For instance, Cocolas et al. (97) also reported that some participants expressed feelings of hypocrisy when encountering difficulties trying to balance their love for travelling with their environmentally responsible values. These similarities within different areas of tourism opens an important avenue for future research to further explore the tensions between environmentally responsible individuals and a sustainable way of, in this case, travelling or adventuring.

In addition, other guides reported feeling emotionally affected by climate change and were surprised when they realised they did not share or discuss their deeper concerns with clients or friends, as Izabella explained: “*I'm curious about how little I share … [about] the wider issues of kind of climate change and those things [but] I think about them quite a lot … [guides] don't like to moan about stuff 'cause we know we've got the best job in the world but genuinely I know that is something that I really gloss over when I talk … “*

As suggested by the data above, guides' emotional responses to climate change was varied and appeared to be linked to how connected they felt to nature. Participants who felt more connected to nature, reported being more psychologically affected by climate change, which manifested as negative emotions and/or ethical dilemmas. These findings aligned with emerging research seeking to understand how emotions derived from climate change affect wellbeing [e.g., (98)], as well as with research exploring how individuals seek to reduce the tension and dissonance between their environmental attitudes and unsustainable practises [e.g., (97)]. As suggested in the current study, adventure guides may be considered vulnerable to negative wellbeing impacts stemming from climate change as they are likely to have a close relationship with nature due to their unique vocation (99). However, further research is needed to better understand the emotional impacts that ecological changes may have on guides' wellbeing, and potential implications for guiding practise.

### Sharing nature connectedness with others

In addition to the themes discussed above, guides also highlighted the importance of sharing their feelings for nature with others. For example: “*I really like meeting new people and sharing my enthusiasm [for nature]… like being outside on my own wouldn't cut it for me … people are as important as the environment*.” (Izabella). Similarly, another guide reported how the guiding role offered the space to positively influence clients: “*I like being in a position of [facilitating life changing experiences], develop people's thinking … and [help them] better themselves”* and explained how enabling clients to feel empowered to do their own autonomous adventures made guiding an enjoyable experience: “*What I like about my job is making that kind of [meaningful] impact into people so that they can get the confidence to go on and visit these wonderful places on their own … to explore the world more confidently”* (Evan).

However, not all clients facilitated a feeling of mutual connexion. Having a shared love of nature and adventure activities with clients was reported as important to positive guiding experiences: “*if [the clients are] here because they enjoy being outside, enjoy the sporty activity … I'm more motivated to work with this kind of people … they make me [feel] comfortable*” (Elliot).

Connecting with clients enhanced the guiding experience and several participants stressed that guiding was not just about safety and teaching hard skills, but also about transmitting their feelings of connexion with nature to others. For example, a guide reported: “*[Being a guide] is more complicated than just going to [guiding] school … the ideal mountain guide that I would like to become …. Is [not] just showing people how to paddle in a kayak or how to … belay … [but also] introduce[s] more nature to people to bring them closer [to it]”* (Isa). Similarly, another guide hoped to have a deeper impact in the wider world as a result of guiding: “*there's hopefully a bigger influence that I can have beyond just teaching people outdoor skills … a bigger political kind of impact*” (Susan).

In line with these data, guides often reported how their guiding enhanced their sense of purpose because they felt they were having a significant impact through their work. For example, Izabella explained: *Knowing that I'm doing something that brings value and worth to people's lives and mine is really important and if … I felt that I was wasting my time and I wasn't doing anything worthwhile then that … would definitely demotivate me*. Similarly, Callum reported: “*I just feel … if you can pass that sort of feeling of connectedness [with nature to clients] and being visible and important as part of universe is really great*” (Callum). Nine participants reported how their guiding role provided a sense of purpose and stressed their desire to help clients develop a sense of nature connectedness that could positively impact the wider world. These data aligned with previous literature that reported how an enhanced a sense of purpose facilitated adventure guide wellbeing. For example, Houge Mackenzie and Raymond (5) suggested that guides gained a sense of purpose by educating clients in relation to environmental protection, as well as by feeling that their job was contributing to the wider world.

The results of the current research suggest that better understanding how to support guides' desires to have a positive impact on the wider world might not only contribute to guide wellbeing but may also support a more ecologically responsible and sustainable future of the adventure industry. These findings compliment emerging research focused on providing specific guidance for adventure tourism operators seeking to develop and a more sustainable global adventure tourism industry (1). However, as suggested by the data above, future research should also aim to provide targeted guidance specifically designed for adventure guides. This will ensure that guide wellbeing is well-supported and potential tensions between environmental attitudes and unsustainable practises are reduced [e.g., (97)].

### Theoretical implications

This research aimed to understand adventure guides' experiences of nature connectedness and to explore potential links between nature connexion, climate change and wellbeing for adventure guides. The findings from this study expand scholarship in adventure tourism and sustainable tourism literature [e.g., (3)], as well as contributing to the emerging field of positive tourism [e.g., (100)]. The study findings highlighted the nuanced and important relationship between adventure guides and the natural environments they operate within. Although guides described having dualistic relationships with nature (59), they also reported a strong connexion with nature characterised by feelings of “oneness” with their natural environments. Interestingly, not all types of nature, nor length of time spent outdoors, accounted for this connexion. Rather, guides identified key factors that facilitated nature connectedness, such as the degree of wilderness and biodiversity present in an environment, and the level of personal responsibility they felt for others' safety and wellbeing (e.g., heightened responsibility for others could reduce attentional focus on and connexion to nature). In addition, the findings highlighted the value that guides placed on sharing their love for nature with clients. Guides explained how educating clients on their natural surroundings and deepening clients' connexion to nature imbued them with a sense of purpose *via* their work. This finding provides empirical support for a recently proposed conceptual model of adventure tour guide wellbeing, particularly in relation to the role of meaning and purpose in guide wellbeing outcomes (5).

The findings also highlighted the importance of further investigating adventure guides' perceptions of, and psychological responses to, climate change and the implications for their wellbeing. For instance, while some guides reported that climate change greatly affected their moods, others reported that climate change had little or no influence on their psychological states or overall wellbeing. These initial findings suggested that guides who reported a stronger sense of connexion with nature may also have a stronger need to care for nature, which in turn may create cognitive dissonance in relation to their professional activities. Specifically, guides reporting strong nature connexion also described an emotional ethical dilemma in relation to attempting to reconcile their love for nature and their desire to transmit environmental values with unsustainable adventure travel practises entailed in their guiding work (e.g., long-haul aviation). These findings should be treated with caution as they are exploratory and reflect a small sample of the diverse adventure guiding population; however, they suggest that understanding the nuanced psychological impacts of climate change for adventure guide wellbeing is an important and fruitful avenue of further research. The findings of the present study suggest that better understanding adventure guides' perceptions and experiences of nature connexion in relation to climate change will (1) expand the extant literature in this area and emerging theorical models of adventure guide wellbeing, and (3) inform practical approaches to supporting guide wellbeing and developing a more sustainable adventure tourism industry.

### Limitations and future directions

This study represents a novel contribution to the literature as it is the first study that explicitly explores adventure guide perceptions of nature connexion in relation to climate change and wellbeing. However, whilst this research sought to include a diverse sample of participants by attempting to recruit guides from different types of adventure activities, sociocultural backgrounds, and genders, the final sample was male and European dominated, and was limited to a subset of adventure activities. Thus, these findings may not reflect perceptions of the larger adventure guiding population, and they are limited by the diversity of participants in relation to activity type, gender, nationality and sociocultural background. These results should therefore be treated as exploratory and a starting point for further research in this domain.

Further research should expand and diversify the sample to include a wider range of activity and guide demographics (e.g., age, gender, length of guiding career, land/air/water-based activities, geographical location, client type). Longitudinal and mixed-methods research (e.g., qualitative, quantitative, ethnographic) on this topic would also be useful in terms of tracking guide wellbeing over time in relation to climate change in specific geographical locations, and identifying mechanisms underlying the relationships proposed in the current study. Future research may also benefit from integrating eco-psychology perspectives, as suggested by Hanna et al. (3), and/or positive psychology perspectives [e.g., (5)], with sustainable adventure tourism literature to further develop empirical and theoretical understandings of this topic.

## Conclusions

The global tourism industry is changing rapidly in the 21st century, and with it the practises and perceptions surrounding adventure tourism and adventure guiding. In addition to pressing environmental concerns related to tourism [e.g., (101)], there is also increasing awareness of the importance of worker wellbeing in the tourism industry [e.g., (102)]. Fostering a more holistic understanding of adventure guide perceptions and experiences is required to ensure that their wellbeing is supported, and that they are equipped for the social and environmental demands of 21st century clients, regulations and environments. Adventure guides have the potential to be catalysts of change and inspire clients to develop a deeper sense of connectedness with nature. Global uncertainties due to the COVID-19 pandemic and rapidly changing geopolitical environments have taken a heavy toll on our collective psychological and planetary wellbeing [e.g., (103)]. Thus, it is timely and relevant to explore key determinants of guide, and client, wellbeing, and practical opportunities for adventure tourism operators, guides, and clients to enhance their wellbeing *via* deep connexions with nature. Adventure guides are well-positioned to facilitate these connexions due to their intimate relationships with nature, which thereby may help them enhance clients' eco-centric perspectives and engage them with climate change issues. This study complements the growing body of research on adventure tourism and wellbeing, and identifies fruitful avenues for further understanding adventure guide wellbeing in relation to nature connexion and climate change.

## Data availability statement

The raw data supporting the conclusions of this article will be made available by the authors, without undue reservation.

## Ethics statement

The studies involving human participants were reviewed and approved by School of Psychology, University of East London. The patients/participants provided their written informed consent to participate in this study.

## Author contributions

EV and SH contributed to conception, design of the study, and organised the database. EV wrote the first draft of the manuscript. Both authors contributed to manuscript revision and approved the submitted version.

## Conflict of interest

The authors declare that the research was conducted in the absence of any commercial or financial relationships that could be construed as a potential conflict of interest.

## Publisher's note

All claims expressed in this article are solely those of the authors and do not necessarily represent those of their affiliated organizations, or those of the publisher, the editors and the reviewers. Any product that may be evaluated in this article, or claim that may be made by its manufacturer, is not guaranteed or endorsed by the publisher.

## Footnotes

^1^In this paper, the term ‘climate change' is used in accordance with the United Nations (n.d.) (2), which applies climate change as an umbrella term that encompasses a range of interrelated environmental crises including deforestatation and the rise of emissions, which have a long term impact in temperatures, and all the consequences derived as a result of this increase (e.g., intense droughts, rising sea levels, loss of biodiversity).
